# Insulin-like growth factor 2 mRNA-binding protein 1 (IGF2BP1) in hematological diseases

**DOI:** 10.1186/s10020-024-00936-2

**Published:** 2024-09-28

**Authors:** Shuangping Ma, Yiran Qin, Wenjie Ren

**Affiliations:** Institutes of Health Central Plains, Xinxiang Medical University, Xinxiang, 453003 China

**Keywords:** IGF2BP1, m6A, Embryogenesis, Β-thalassemia, SCD

## Abstract

The oncofetal mRNA-binding protein IGF2BP1 belongs to a conserved family of RNA-binding proteins. It primarily promotes RNA stability, regulates translation and RNA localization, and mediates gene expression through its downstream effectors. Numerous studies have demonstrated that IGF2BP1 plays crucial roles in embryogenesis and carcinogenesis. IGF2BP1-modulated cell proliferation, invasion, and chemo-resistance in solid tumors have attracted researchers’ attention. Additionally, several studies have highlighted the importance of IGF2BP1 in hematologic malignancies and hematological genetic diseases, positioning it as a promising therapeutic target for hematological disorders. However, there is a lack of systematic summaries regarding the IGF2BP1 gene within the hematological field. In this review, we provide a comprehensive overview of the discovery and molecular structure of IGF2BP1, along with recent studies on its role in regulating embryogenesis. We also focus on the mechanisms by which IGF2BP1 regulates hematological malignancies through its interactions with its targeted mRNAs. Furthermore, we systematically elucidate the function and mechanism of IGF2BP1 in promoting fetal hemoglobin expression in adult hematopoietic stem/progenitor cells. Finally, we discuss the limitations and challenges of IGF2BP1 as a therapeutic target, offering insights into its prospects.

## Introduction

It is well-known that insulin-like growth factor 2 binding protein 1 (IGF2BP1) is an “oncofetal” protein specially expressed in a broad range of fetal tissues and many cancers. This gene is involved in the transport, translation, localization, and stability of certain mRNAs which play vital roles in embryogenesis and carcinogenesis (Degrauwe et al. 2016; Fakhraldeen et al. 2015; Mahaira et al. 2014). Studies have shown that IGF2BP1 plays important roles in regulating mRNA targets, such as *PTEN*, *ACTB*, *MAPK4*, *c-MYC*, *CD44*, etc., especially in an N6-methyladenosine (m^6^A)-dependent manner (Mongroo et al. 2011; Nicastro et al. 2017; Samuels et al. 2020). In 2000, Doyle et al. found that the IGF2BP1 gene accelerated breast cancer by upregulating c-MYC abundance (Doyle et al. 2000). Subsequently, it was reported that IGF2BP1 was overexpressed in many cancers, particularly in colorectal cancer (Kuhn et al. 2022; Ross et al. 2001), hepatocellular carcinoma (Cai et al. 2021), gallbladder cancer (Kessler et al. 2017), and breast cancer (Wang et al. 2023b; Zhu et al. 2021), and is associated with a poor clinical outcome and shorter survival. Recently, it was widely reported that IGF2BP1 as a m^6^A “reader” involved in tumor immune microenvironment (TME) regulation activated immune cell infiltration and promoted cancer development by recognizing m6A target transcripts (Elcheva et al. 2023; Liu et al. 2022a, b). Strikingly, Glaß and Hüttelmaier showed that the recruitment of IGF2BP1 promoted the transmission of hepatitis B/C and human papillomaviruses (Glass and Huttelmaier 2023), which suggested that IGF2BP1 had various functions in different fields. In addition to participating in post-transcriptional modifications, the transcription of IGF2BP1 is often regulated by RNA polymerases and transcription factors (Table 1). It was uncovered that RNA polymerase II elongation regulates IGF2BP1 gene expression in a pattern of allelic bias (Thomas et al. 2011). TAZ as an enhancer promoted IGF2BP1 expression in mesenchymal glioblastoma (Yang et al. 2023). Forkhead Box A2 (FoxA2) as a transcription repressor binding to the proximal promoter of IGF2BP1 inhibited pyroptosis in human endometrial stromal cells (HESC) (Feng et al. 2023b). Another study showed that ligustrazine exerted the anti-inflammatory ability via STAT3 binding to IGF2BP1 promoter, and IGF2BP1 maintaining the stability of RELA mRNA (Feng et al. 2023a).

**Table 1:** Molecules that regulate IGF2BP1

Molecules	Binding sites on IGF2BP1	Regulation styles	References
RNA polymerase II	promoter	Promoting transcription	(Thomas et al. 2011)
TAZ	enhancer	Promoting transcription	(Yang et al. 2023)
FoxA2	promoter	Repressing transcription	(Feng et al. 2023b)
STAT3	promoter	Promoting transcription	(Feng et al. 2023a)
MYCN	E-Boxes	Promoting transcription	(Hagemann et al. 2023)
c-myc	-	Promoting transcription	(Noubissi et al. 2010)
β-catenin	Promoter	Promoting transcription	(Gu et al. 2008; Noubissi et al. 2009)
HIF1α	Promoter	Promoting transcription	(An et al. 2023; Craig et al. 2012)
TFAP4	Promoter	Promoting transcription	(Shen et al. 2022b)
KDM5B	Promoter	Promoting transcription	(Du et al. 2023)
TET1	Promoter	Promoting transcription	(Mahaira et al. 2014)
MIF	promoter	Promoting transcription	(Mao et al. 2023)
LTN1	protein	Promoting ubiquitination	(Peng et al. 2023)
PRMT3	protein	Arginine methylation	(Shi et al. 2023b)
USP10	Protein	Deubiquitinating	(Shi et al. 2023a)
TRIM29	Protein	Promoting ubiquitination	(Jiang et al. 2024)
FBXO45	Protein	Promoting ubiquitination	(Lin et al. 2021b)
METTL21C	Protein	Lysine trimethylation	(Wang et al. 2023c)

Leukemia is a group of diseases characterized by clonal proliferation of abnormal hematopoietic cells leading to disruption of normal marrow function and marrow failure, and the precise location of origin is usually unknown (Whiteley et al. 2021). Acute myeloid leukemia (AML), Acute lymphoblastic leukemia (ALL), chronic myeloid leukemia (CML), and chronic lymphocytic leukemia (CLL) are four common types of leukemia. As early as 2005, the insulin-like growth factor 2 mRNA binding protein 3 (IGF2BP3) was identified to promote the proliferation of human K562 leukemia cells by targeting IGF2 leader-3 mRNA (Liao et al. 2005). Since then, IGF2BPs in liquid tumors have been valued for their unique regulatory roles, especially IGF2BP1. Importantly, de Vasconcellos claimed that IGF2BP1 has extraordinary potential in enhancing γ-globin expression in human erythroblasts and become a promising therapeutic target for β-hemoglobinopathies (de Vasconcellos et al. 2017). In this paper, we systematically summarized the studies on IGF2BP1 in recent years and elucidated the mechanism of IGF2BP1 in regulating embryogenesis, hematologic malignancies, and hematologic genetic diseases, providing some clues for the diseases that target IGF2BP1. Finally, we discussed the limitations of IGF2BP1 as a potential therapeutic target for hematological diseases.

## IGF2BPs: the important molecules involved in post-transcription regulation

### The discovery process of IGF2BPs

**Fig. 1:**
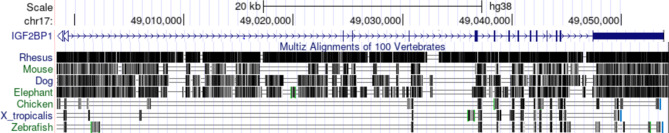
IGF2BP1 conservation among mammals from UCSC Genome Browser

**Fig. 2:**
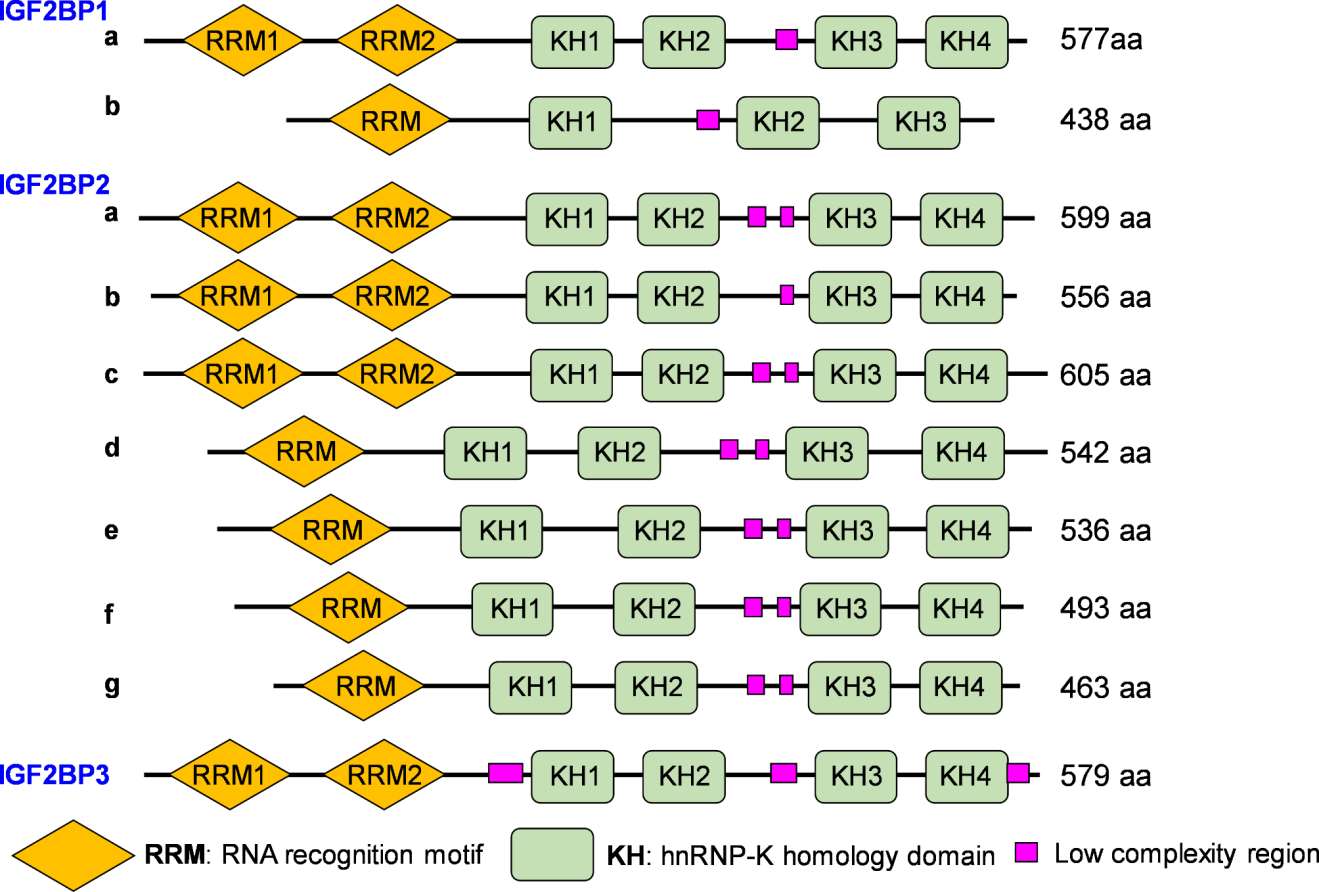
The protein structures of human IGF2BPs. IGF2BP1 has two isoforms. IGF2BP1-a variant (NM_006546) represents the longer transcript and encodes the longer isoform. IGF2BP1-b variant (NM_001160423) lacks two alternate in-frame exons compared to IGF2BP1-a. IGF2BP2 has a total of seven isoforms. IGF2BP2-a (NM_006548) and IGF2BP2-c (NM_001291869) are the two longer isoforms. IGF2BP2-b (NM_001007225) lacks an alternate in-frame exon compared to IGF2BP2-a. IGF2BP2-d- IGF2BP2-g are shorter isoforms that lack an RRM domain. IGF2BP3 has only one variant

The insulin-like growth factor 2 mRNA binding proteins (IGF2BPs), comprising IGF2BP1, IGF2BP2, and IGF2BP3, belong to a conserved family of single-stranded RNA binding proteins. These proteins were first identified in 1999 for their ability to bind to the 5’-UTR of IGF2 mRNA (Nielsen et al. 1999). The earliest discovery was IGF2BP1, which was found to bind to the coding region of c-MYC, thereby stabilizing its mRNA (Bernstein et al. 1992). IGF2BP1 is also known by several other names, including IMP1, ZBP1, VICKZ1, and CRD-BP. Intriguingly, an evolutionary analysis of the IGF2BP1 nucleotide sequence using the UCSC database has revealed its high conservation among mammals such as rhesus, mouse, dog, and elephant, but lower conservation in species like chicken and zebrafish (Fig. 1). In 1999, Zhang et al. reported that IGF2BP2 was present as an intracellular antigen in 30–40% of hepatocellular carcinoma patients (Lederer et al. 2014; Zhang et al. 1999). Unlike IGF2BP1 and IGF2BP3, IGF2BP2 features more splicing variants and exhibits unique characteristics in biological regulation. IGF2BP3, also known as a KOC protein, is highly overexpressed in pancreatic cancer tissues and various other tumors (Mueller-Pillasch et al. 1997). As highly conserved RNA-binding proteins, IGF2BPs play crucial roles in RNA splicing, ribosome translation, RNA decay, and mRNA stability (Degrauwe et al. 2016). Among the three IGF2BPs, IGF2BP1 and IGF2BP3 share a significant similarity, with about 73.2% similarity in their amino acid sequences (Fig. 2; Table 2**)**. IGF2BPs are widely expressed in most tissues and organs during embryogenesis. However, in adult tissues, IGF2BP2 is particularly ubiquitous, especially within the gastrointestinal digestive system (Bell et al. 2013; Lederer et al. 2014).

### Molecular structure of IGF2BPs

Generally, IGF2BPs are composed of two RNA recognition motifs (RRMs) and four hnRNP-K homology (KH) domains (Fig. 2). IGF2BP1, as a typical example, relies heavily on its KH1/2 domains to maintain the stability of IGF2BP1-RNA complexes. In 1995, Kiledjian et al. first reported that proteins containing KH domains could regulate mRNA stability (Kiledjian et al. 1995). For instance, the KH1/2 domains of IGF2BP1 modulated the binding to the cis-elements of ACTB 3’-UTR (Bell et al. 2013). Interestingly, KH1/2 domains also mediated the binding of IGF2BP1 to the c-MYC coding region stability determinant (CRD) in vitro. With the development of biotechnology, the mechanisms by which IGF2BP1 stabilizes RNA complexes have become clearer. RRMs enhance the stability of the IGF2BP1-RNA complex in a targeted manner, while KH domains facilitate the interaction between IGF2BP1 and RNAs (Dagil et al. 2019; Hollingworth et al. 2012). Strikingly, Huang et al. claimed that IGF2BPs stabilized c-MYC mRNAs mainly through the KH3 and KH4 domains and through m6A modifications (Huang et al. 2018).

**Table 2:** The amino acid similarity of human IGF2BPs

	Amino acid similarity	Species
IGF2BP1^*^ vs. IGF2BP2^#^	65.61%	Homo species
IGF2BP1 vs. IGF2BP3	73.2%	Homo species
IGF2BP2 vs. IGF2BP3	63.25%	Homo species

*IGF2BP1 is the IGF2BP1-a. #IGF2BP2 is the IGF2BP2-a

### Role of IGF2BP1 in embryogenesis

The critical role of IGF2BP1 in embryonic development is well-established. It is highly expressed in fertilized eggs and embryos, but its expression is almost negligible in normal adult tissues (Bell et al. 2013). Research on embryos from Xenopus, zebrafish, and mice revealed that IGF2BP1 is present in various cell types, including migratory neural ridges, gill arches, and cranial neural ridges (CNC) (Yaniv et al. 2003). Consistent with these findings, Bell et al. reported that in 16-week-old adult mice, IGF2BP1 showed slight expression in the lung, brain, and spleen, but was undetectable in other organs according to semi-quantitative RT-PCR analysis (Bell et al. 2013). Furthermore, Hammer et al. found that IGF2BP1 expression was significantly higher in the gonads of adult mice and humans compared to the prostate, trachea, and kidney in adult humans (Hammer et al. 2005). The elevated levels of IGF2BP1 in the testis may play a role in maintaining the activity of spermatogonial stem cells or could be involved in germ-cell movement during spermatogenesis.

The presence of IGF2BP1 is indispensable for the normal progression of embryogenesis. Mice deficient in IGF2BP1 suffer from dwarfism, poor survival ability, and intestinal dysplasia. Consequently, IGF2BP1-deletion mice have a perinatal mortality rate of 60%. Even if these mice survive birth, they exhibit significantly smaller bodies compared to wild-type mice and produce abnormal tissues and organs during embryogenesis (Hansen et al. 2004). In addition to its critical role in embryogenesis, the SNP rs9674544 in IGF2BP1 was found to be significantly associated with deciduous tooth development in infancy (Pillas et al. 2010). Moreover, IGF2BP1 was also involved in the development of the central nervous system (Nunez et al. 2022). Specifically, the production of cerebral cortex was disorganized in IGF2BP1-knockout mice during embryogenesis, and the cell density of the cortical limbic region was also decreased at E17.5. This was mainly because IGF2BP1 deficiency downregulated its target actin expression, severely hindering cell migration and orientation (Nunez et al. 2022).

Additionally, during neuronal development, IGF2BP1 partially regulated neurite growth, neuronal cell migration, and axon guidance by controlling the spatiotemporal activation of protein synthesis, such as ACTB mRNA (Fabrizio et al. 2008; Perycz et al. 2011). IGF2BP1 controlled the subcellular localization of ACTB in primary fibroblasts and neurons by binding to the cis-element of ACTB 3 ‘-UTR (Gong et al. 2016). Furthermore, IGF2BP1 was involved in determining the cell fate of testicular stem cells and mediated neuronal differentiation and maturation of the nervous system during regeneration (Boylan et al. 2008; Donnelly et al. 2011; Toledano et al. 2012). One study showed that the low expression of IGF2BP1 in the dorsal neural tube was necessary for CNC delamination and migration, however, inhibiting its expression enhanced CNC delamination and induced epithelial cell segregation. Due to its interaction with ITG6 mRNA, IGF2BP1 was negatively correlated with epithelial-to-mesenchymal transition and played a crucial role in maintaining epithelial integrity (Carmel et al. 2015). In another study focused on neural crest migration in amphibians, downregulation of IGF2BP1 expression using antisense morpholino oligonucleotides (AMO) in embryos resulted in an increased CNC migration rate (Yaniv et al. 2003). These studies highlight the significant roles of IGF2BP1 in regulating cell growth and differentiation during organismal development, suggesting that aberrant expression of this gene may lead to tissue and organ dysplasia through the dysregulation of its target proteins, such as ACTB and ITGA6 (Table 3).

**Table 3:** Target mRNAs of IGF2BP1

Target	Binding sites		Regulation styles		References	
IGF2	5′-UTR		Inhibiting mRNA translation		(Nielsen et al. 1999)	
ACTB	3′-UTR		Inhibiting mRNA translation		(Huttelmaier et al. 2005; Leung et al. 2006; Yao et al. 2006)	
ACTB	3′-UTR		mRNA transport		(Eom et al. 2003; Nicastro et al. 2017)	
c-MYC	CDS		Inhibiting mRNA decay		(Bernstein et al. 1992; Zhu et al. 2021)	
NF-κB	CDS		Improving p65-p52 nuclear translocation		(Xie et al. 2019a)	
BZW2	CDS		Maintaining mRNA stability		(Long et al. 2023)	
ETV6::RUNX1	CDS		Maintaining mRNA stability		(Sharma et al. 2023)	
BCL11A	CDS		Inhibition of mRNA translation		(de Vasconcellos et al. 2017)	
HOXB4	CDS		Maintaining mRNA stability		(Elcheva et al. 2020)	
MYB	CDS		Maintaining mRNA stability		(Elcheva et al. 2020)	
ALDH1A1	CDS		Maintaining mRNA stability		(Elcheva et al. 2020)	
HOXB2	CDS		Maintaining mRNA stability		(Elcheva et al. 2020)	
FSCN1	CDS		Maintaining mRNA stability		(Xie et al. 2021)	
TK1	CDS		Maintaining mRNA stability		(Huang et al. 2018)	
MARCKSL1	CDS		Maintaining mRNA stability		(Huang et al. 2018)	
E2F1	3′-UTR		Maintaining mRNA stability		(Mao et al. 2023)	
MGAT5	CDS		Maintaining mRNA stability		(Yang et al. 2021)	
PDLIM7	3′-UTR		Maintaining mRNA stability		(Muller et al. 2019)	
FOXK1	3′-UTR		Maintaining mRNA stability		(Muller et al. 2019)	
CPT1A	CDS, 3’-UTR		Maintaining mRNA stability		(Shi et al. 2023a)	
PEG10	3′-UTR		Maintaining mRNA stability		(Zhang et al. 2021a)	
MKK6	3′-UTR		Maintaining mRNA stability		(Zhao et al. 2023)	
MAPK14	3′-UTR		Maintaining mRNA stability		(Zhao et al. 2023)	
PKM2	3′-UTR		Maintaining mRNA stability		(Lv et al. 2023)	
SMAD1	3′-UTR		Maintaining mRNA stability		(Huang and Wang 2022)	
JAK2	CDS		Maintaining mRNA stability		(Dong et al. 2021)	
SEMA3A	-		Maintaining mRNA stability		(Dhamdhere et al. 2023)	
EZH2	-		Maintaining mRNA stability		(Sperling et al. 2022)	
SIK2	-		Maintaining mRNA stability		(Xu et al. 2023)	
MDM2	3′-UTR		Maintaining mRNA stability		(Mu et al. 2022)	
INHBA	-		Maintaining mRNA stability		(Wang et al. 2023a)	
KIF2A	-		Maintaining mRNA stability		(Sun et al. 2023)	
TK1	-		Maintaining mRNA stability		(Shen et al. 2022a, b)	
MIR210HG	3′-UTR		Maintaining mRNA stability		(Shi et al. 2022)	
Netrin-1	-		Maintaining mRNA stability		(Zhang et al. 2020)	
Gbp 11	3′-UTR		Maintaining mRNA stability		(Ding et al. 2022)	
Cp	3′-UTR		Maintaining mRNA stability		(Ding et al. 2022)	
IQGAP3	3′-UTR		Maintaining mRNA stability		(Myint et al. 2022)	
SEMA3A	CDS		Maintaining mRNA stability		(Dhamdhere et al. 2023)	
SHMT2	CDS		Maintaining mRNA stability		(Dhamdhere et al. 2023)	
PD-L1	3′-UTR		Maintaining mRNA stability		(Jiang et al. 2024)	
BRTC	CDS		Maintaining mRNA stability		(Dhamdhere et al. 2023)	
PTBP1	-		Positive correlation		(Su et al. 2024)	
ERK2	CDS		Maintaining mRNA stability		(Bley et al. 2021)	
SRC	CDS		Maintaining mRNA stability		(Bley et al. 2021)	
E7	3′-UTR		Maintaining mRNA stability		(Wang et al. 2022a)	
SLC7A11	3′-UTR		Maintaining mRNA stability		(Liu et al. 2022a)	
ERRα	3′-UTR		Inhibition of mRNA decay		(He et al. 2022)	
MIR210HG		3′-UTR	Maintaining mRNA stability	(Shi et al. 2022)		
CD47	-		Maintaining mRNA stability		(Fan et al. 2021)	
Slug	3′-UTR		Maintaining mRNA stability		(Zhong et al. 2023)	
RUNX1	-		Maintaining mRNA stability		(Liu et al. 2022b)	
MMP3	3′-UTR		Maintaining mRNA stability		(Zhao et al. 2024b)	
DHAV-1	3′-UTR		Promoting translation		(Chen et al. 2019)	
eIF4G	3′-UTR		Promoting translation		(Song et al. 2023)	
ABL	KH1/2 domain		Maintaining mRNA stability		(Wang et al. 2022b)	
Gli1	-		Promoting translation		(Meng et al. 2023)	
HMGB1	3′-UTR		Maintaining mRNA stability		(Liang et al. 2023)	
BUB1B	-		Maintaining mRNA stability		(Hu et al. 2023)	
MAPK4	-		Repressing mRNA translation		(Stohr et al. 2012)	
PTEN	-		Maintaining mRNA stability		(Stohr et al. 2012)	
LDHA	3′-UTR		Maintaining mRNA stability		(Zhang et al. 2021b)	
IL11	CDS		Maintaining mRNA stability		(Wei et al. 2023)	
MCAM	-		Promoting m6A methylation		(Song et al. 2024)	
GPX4	CDS, 3′-UTR		Maintaining mRNA stability		(Wang et al. 2023a)	
DDX27	-		Maintaining mRNA stability		(Chen et al. 2023)	
HCV	5′-UTR, 3′-UTR		Promoting translation		(Weinlich et al. 2009)	
CCN1	3′-UTR		Maintaining mRNA stability		(Rana et al. 2024)	
HDAC4	3′-UTR		Maintaining mRNA stability		(Shen et al. 2022a)	
LEF1	3′-UTR		Maintaining mRNA stability		(Zirkel et al. 2013)	
FNDC3B	-		Maintaining mRNA stability		(Li et al. 2023)	
PCAT6	-		Maintaining mRNA stability		(Liu et al. 2020)	
Ptgs2	KH domain		Maintaining mRNA stability		(Manieri et al. 2012)	
TRPV1	-		Maintaining mRNA stability		(Bai et al. 2024)	
SOX2	-		Maintaining mRNA stability		(Huang et al. 2024)	
ZHX2	-		Maintaining mRNA stability		(Xiao et al. 2024)	
RPL36	-		Attenuating mRNA stability		(Wang et al. 2024)	
CCL5	3′-UTR		Maintaining mRNA stability		(Zhao et al. 2024a)	
YES1	3′-UTR		Maintaining mRNA stability		(Cai et al. 2021)	
FOXM1	-		Maintaining mRNA stability		(Leclair et al. 2024)	
STUB1	-		Maintaining mRNA stability		(Tang et al. 2023)	
HEG1	-		Maintaining mRNA stability		(Shi et al. 2023b)	

Until now, the molecular mechanisms involving IGF2BP1 in embryonic development have not been fully elucidated. It is known that parthenogenesis can be activated by aberrant epigenetic modifications, such as m6A. In mouse parthenogenetic embryos, the downregulation of IGF2BP1 significantly reduced cleavage and blastula rates (Hao et al. 2020). Meanwhile, the expression level of m6A was also significantly decreased upon IGF2BP1 downregulation (Hao et al. 2020). IGF2BP1 was targeted by microRNA-670, and the inhibition of microRNA-670 not only facilitated IGF2BP1 expression but also improved cleavage and blastula rates (Hao et al. 2020). Researchers explored the mRNA expression of IGF2BP1 in white Muscovy duck embryos at different developmental stages and found that its mRNA expression increased at E13 and E17, then rapidly decreased at E21, reaching the lowest level at E25, before increasing again at E29. This indicates a turnover overall expression pattern of IGF2BP1 during the embryogenesis of white Muscovy ducks (Tao et al. 2022). Interestingly, the expression of IGF2BP1 varied between sexes; for example, the mRNA expression level of IGF2BP1 in male white Muscovy ducks was markedly higher than that in females at E17 (Tao et al. 2022). This difference might be related to the specific high expression of IGF2BP1 in the testis compared to the placenta (Hammer et al. 2005). A novel conserved lncRNA, THOR (ENSG00000226856), exhibited widespread expression during early development in zebrafish and mice while displaying testicle-specificity in adult tissues of humans, zebrafish, and mice. It was demonstrated that THOR interacted with IGF2BP1 across multiple tissues (Wang et al. 2019; Wu et al. 2021; Xue et al. 2019), suggesting that IGF2BP1 may play a role in the development of sexual organs.

## Role of IGF2BP1 in hematological diseases

### Role of IGF2BP1 in hematological malignancies

A large body of evidence has demonstrated that IGF2BP1 is involved in cancer metastasis and proliferation, making it a potential therapeutic target. Notably, IGF2BP1 promotes tumor occurrence and progression in an m6A-dependent manner, which is particularly remarkable (Sun et al. 2022). IGF2BP1 is a unique m6A recognition protein, with its KH3/KH4 domains responsible for recognizing m6A sites on target mRNAs. This recognition promotes the stability of target mRNAs and enhances translation efficiency in an m6A-dependent manner (Huang et al. 2018). Studies on IGF2BP1 have primarily focused on solid tumors, often overlooking its essential role in hematological malignancies. The malignant potential of cancer cells can be controlled by inducing their differentiation or inhibiting their growth. Many blood cancers, like leukemia, originate in the bone marrow and produce large numbers of aberrant blood cells, leading to increased risks of infection, bone pain, fatigue, bleeding, and bruising (Jamal et al. 2023). LIN28/LIN28B is highly expressed in many solid tumors as well as hematological malignancies. These proteins function as stem cell reprogramming factors, downregulating the let-7 family of microRNAs and promoting cancer stem cell differentiation (Su et al. 2012; Zhou et al. 2013). It has been observed that IGF2BP1 is widely overexpressed in advanced leukemia (Alam et al. 2015) and can inhibit the differentiation of cancer stem cells (Ali Hosseini Rad et al. 2013; Viswanathan et al. 2009; Yang et al. 2010). Therefore, IGF2BP1 plays a role in maintaining the properties of leukemia stem cells by regulating transcriptional and metabolic pathways (Elcheva et al. 2020). Moreover, silencing IGF2BP1 expression induced G2/M arrest, repressing the proliferation and colony formation of AML cells. Importantly, IGF2BP1 had been identified as a downstream effector of LIN28B by let-7 miRNA, and its expression was downregulated when LIN28B was inhibited. Consequently, LIN28B overexpression indirectly increased IGF2BP1 expression by inhibiting let-7 miRNAs in AML cells (Zhou et al. 2017).

More than that, Xie et al. showed that IGF2BP1 played a key role in the LPS-induced signaling pathway and inflammatory response. Specifically, IGF2BP1 stimulated the release of inflammatory factors by activating the NF-KB signaling pathway (Xie et al. 2019a, b). Furthermore, a novel IGF2BP1-binding lncRNA, LIN28B-AS1, has been shown to enhance NF-κB activation and augment the production of pro-inflammatory cytokines induced by lipopolysaccharide (LPS) in THP-1 macrophages (Xie et al. 2019b). Elcheva et al. reported that silencing IGF2BP1 impaired the proliferation and tumorigenesis of leukemia cells. IGF2BP1 inhibition also facilitated the sensitivity of leukemia cells to chemotherapy drugs (Elcheva et al. 2020).

Recently, several noncoding RNAs have been reported to mediate cancer progression by binding to the 3’UTR or KH domains of IGF2BP1 (Table 4). For instance, hsa-circ_0003420 repressed IGF2BP1 expression by targeting the 3 ‘-UTR of IGF2BP1, leading to growth arrest and apoptosis of AML cells (Lin et al. 2021a). Recent studies have found that lncRNA FEZF1-AS1 was significantly overexpressed in both tissues and cell lines of multiple myeloma. This overexpression promoted the proliferation and inhibited apoptosis of myeloma cells by stabilizing BZW2 mRNA through its interaction with IGF2BP1 (Long et al. 2023). Furthermore, 2-[(5-bromo-2-thienyl) methylene] amino benzamide (BTYNB) had been identified as a small molecule inhibitor of IGF2BP1. Based on a fluorescence anisotropy assay, BTYNB was screened out from a library of 160,000 small molecules. This compound specifically targeted c-Myc mRNA, downregulated β-TrCP1 mRNA, reduced NF-κB activity, and inhibited the proliferation and protein synthesis of IGF2BP1-positive tumor cells (Mahapatra et al. 2017). In addition, it was found that BTYNB induced the S phase arrest and facilitated the apoptosis and differentiation of leukemia cells by blocking IGF2BP1 expression, ultimately slowing down the progression of leukemia (Jamal et al. 2023). Although many specific IGF2BP1 small molecule inhibitors have been recently identified in solid cancers, few were applied to hematological malignancies (Shang et al. 2023; Singh et al. 2024; Wallis et al. 2022). Notably, IGF2BP1 is highly expressed in t(12; 21)(p13; q22)-positive ALL. To investigate the regulatory mechanism of IGF2BP1 in t(12; 21)(p13; q22)-positive ALL, IGF2BP1 was stably knocked down in the REH cell line. Compared to the control cell line, the proliferation capacity of REH cells was significantly impeded. Meanwhile, spontaneous cell mortality increased as the mRNA level of STAT3 was markedly reduced due to IGF2BP1 downregulation (Stoskus et al. 2016a). IGF2BP1 targets ETV6/RUNX1, and the stability of its transcript mediates leukemogenesis in t(12; 21)(p13; q22)-positive ALL (Stoskus et al. 2016b). Consistent with the above studies, IGF2BP1 was found to be particularly overexpressed in ETV6::RUNX1 chromosomal translocation-positive childhood leukemia in an Indian cohort (Sharma et al. 2023). This overexpression is crucial for the survival of ETV6::RUNX1-positive B-ALL cells. Mechanically, IGF2BP1 activates the TNF-α/NF-κB and PI3K-Akt signaling pathways primarily by binding and stabilizing the fused transcript of ETV6::RUNX1, as revealed by transcriptome sequencing analysis (Sharma et al. 2023).

**Table 4:** Noncoding RNAs that regulate IGF2BP1

Noncoding RNAs	Binding sites on IGF2BP1		Regulation styles	References
Let-7 family	3’UTR	Posttranscriptional repression		(Cheng et al. 2013)
circPTPRA	KH3/4 domains	Translational suppression		(Xie et al. 2021)
circ_0003420	3’UTR	Posttranscriptional repression		(Lin et al. 2021a)
FEZF1-AS1	-	Positive correlation		(Long et al. 2023)
EMX2OS	-	Translational suppression		(Zhang et al. 2023)
miR-124-3p	3’UTR	Posttranscriptional repression		(Li 2022; Wang et al. 2018)
miR-491-5p	3’UTR	Posttranscriptional repression		(Gong et al. 2016)
miR-506	3’UTR	Posttranscriptional repression		(Luo et al. 2015)
miR-873	3’UTR	Posttranscriptional repression		(Wang et al. 2015)
miR-494	3’UTR	Posttranscriptional repression		(Ohdaira et al. 2012; Wan et al. 2019)
miR-625	3’UTR	Posttranscriptional repression		(Zhou et al. 2015)
miR-150	3’UTR	Posttranscriptional repression		(Qu et al. 2016; Xu et al. 2020)
miR-196b	3’UTR	Posttranscriptional repression		(Rebucci et al. 2015)
miR-1275	3’UTR	Posttranscriptional repression		(Fawzy et al. 2015)
miR-708	3’UTR	Posttranscriptional repression		(Qin et al. 2017)
miR-140-5p	3’UTR	Posttranscriptional repression		(Su et al. 2016)
miR-98-5p	3’UTR	Posttranscriptional repression		(Wang et al. 2020)
miR-186	3’UTR	Transcriptional repression		(Habashy et al. 2022)
miR-4500	3’UTR	Posttranscriptional repression		(Li et al. 2019)

### Role of IGF2BP1 in hematological genetic diseases

**Fig. 3:**
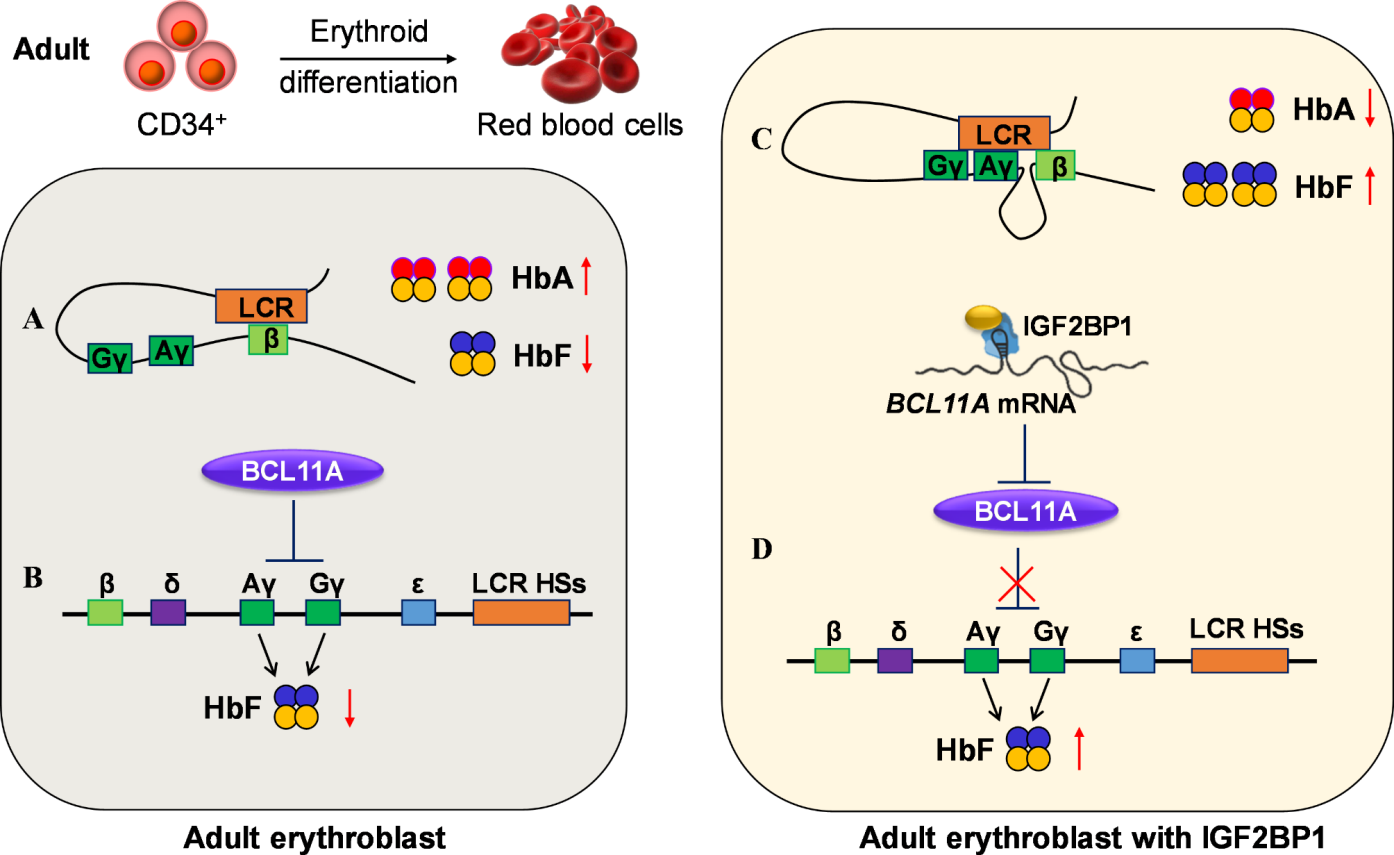
Mechanism diagram of IGF2BP1 regulating hemoglobin conversion

Sickle cell anemia (SCD) and β-thalassemia are both β-hemoglobinopathies caused by the reduction or absence of β-globin chains, leading to ineffective erythropoiesis. This disease is a common inherited hematological disorder worldwide. Increasing the production of fetal hemoglobin (α_2_γ_2_, HbF) is an effective strategy to ameliorate the severity of β-hemoglobinopathies (Basak et al. 2020). Currently, there are four main strategies to promote the synthesis of HbF in sickle cell anemia and β-thalassemia: (1) Decrease or silence the expression of transcriptional repressors. By using siRNA or gene editing techniques to target repressors such as BCL11A and ZBTB7A/LRF, the inhibition of HbF expression can be relieved (Liu et al. 2018; Wu et al. 2019). (2) Disrupt the binding sites of BCL11A and ZBTB7A/LRF. Gene editing can be used to disrupt these binding sites on the γ-globin promoter, enabling the γ-globin promoter to come into contact with the locus control region (LCR) (Metais et al. 2019). (3) Chemical induction of γ-globin production. Various chemical compounds, such as the demethylating agent 5-azacitidine and derivatives of small-chain fatty acids like arginine butyrate, can be used to induce γ-globin production (Rahimmanesh et al. 2022); (4) Epigenetic regulation. This includes the use of deacetylase activators like SRT2104 and SRT1720 (Dai et al. 2017) or leveraging a missense mutation (c.2633G > A, S878F) in the DNMT1 bromo-adjacent homology-1 (BAH1) domain to enhance HbF synthesis (Gong et al. 2021). These strategies collectively represent promising avenues for increasing HbF production and mitigating the effects of β-hemoglobinopathies. As stated above, IGF2BP1 is a fetal-specific expression factor. It has been reported that IGF2BP1 positively regulates the expression of HbF when lentivirus-containing IGF2BP-expressed cassettes are delivered to CD34^+^ cells (Chambers et al. 2020; de Vasconcellos et al. 2017). Overexpression of IGF2BP1 in CD34^+^ cells from SCD and β-thalassemia patients significantly increased HbF expression, reduced the imbalance of α and β-like chains, and alleviated the severity of these diseases (Chambers et al. 2020) (Fig. 3C). The mechanism by which IGF2BP1 promotes HbF expression may involve two aspects: (1) IGF2BP1 overexpression obstructs the translation of BCL11A, significantly alleviating the inhibitory effect of BCL11A on HbF expression (de Vasconcellos et al. 2017) (Fig. 3D); (2) The overexpression of IGF2BP1 facilitates the binding of the γ-globin promoter to the LCR region while partially unbinding the β-globin promoter from the LCR region, thereby promoting the conversion of β-globin to γ-globin (Chambers et al. 2020) (Fig. 3C). In a recent study, a novel HbF suppressor gene, polycombs BMI1, was identified. Knockout of BMI1 in HUDEP-2 cells using CRISPR/Cas9 technology did not affect the expression of adult hemoglobins (α_2_β_2_, HbA, and α_2_δ_2_, HbD), while the expression of fetal globin HBG1/2 was upregulated by 16-folds (Qin et al. 2023). CpG islands adjacent to the promoters of the RNA-binding proteins IGF2BP1, IGF2BP3, and LIN28B were demethylated following BMI1 knockout. Hence, it is considered that BMI1 indirectly raised HbF expression by upregulating IGF2BP1, IGF2BP3, and LIN28B (Qin et al. 2023). Together, these studies suggest that IGF2BP1 has great potential in the treatment of β-hemoglobinopathies.

In healthy adult erythroblast, IGF2BP1 expression is generally at a very low level. (**A**) The adult hemoglobin (HbA) is largely expressed via LCR enhancers in contacting with the β-globin promoter while the fetal hemoglobin (HbF) is slightly expressed in adult erythroblast. (**B**) BCL11A inhibits the HbF expression by repressing the transcription of ^A^γ and ^G^γ in adult erythroblasts. (**C**) When IGF2BP1 is overexpressed in adult erythroblast, LCR looping slides towards the promoters of ^A^γ and ^G^γ and keeps away from the β-globin promoter, thus HbA expression is dramatically decreased and HbF expression is markedly increased. (**D**) The translation of BCL11A mRNA is impeded via binding to IGF2BP1 protein, leading to the reactivation of HbF expression in adult erythroblasts.

## Conclusion

As mentioned above, IGF2BP1 is highly conserved across mammals (Fig. 1) and is widely expressed during the embryonic stage and in various tumor tissues. Literature indicates that IGF2BP1 is involved in the development of fertilized eggs, CNS, and gonads, and it may play a role in sex determination.

IGF2BP1 promotes the proliferation, survival, and invasion of tumor cells through both m6A-dependent and non-m6A-dependent mechanisms. It also maintains tumor stem cell properties, which are closely associated with poor prognosis and metastatic tumors. It has been confirmed that IGF2BP1 plays a dual role in tumor regulation. For instance, in colon stromal cells, IGF2BP1 functions as a tumor suppressor, and its deletion leads to elevated hepatocyte growth factor levels in the microenvironment (Hamilton et al. 2015). It is noteworthy that the tumorigenic driving effect of IGF2BP1 in leukemia is predominantly regulated through non-m6A-dependent mechanism, according to a large number of papers related to hematologic tumors. Whether IGF2BP1 regulation of leukemia is dependent on m6A modification remains unclear and warrants further investigation. The IGF2BP1 inhibitor BTYNB has been shown to effectively repress the progression of leukemia cells, suggesting potential applicability to other tumors. As a promising molecular target for tumors, several small-molecule inhibitors targeting IGF2BP1 have been discovered through high-throughput screening, offering hope for prolonging patient survival.

In this paper, we paid attention to the relationship between IGF2BP1 and the hematopoietic system. In 2005, Ioannidis et al. demonstrated that IGF2BP1 was expressed in umbilical cord CD34^+^ hematopoietic stem cells, but not in cord blood, bone marrow (BM), and peripheral blood cells (Ioannidis et al. 2005). Interestingly, when the DNA methyltransferase inhibitor 5’-azacytidine was added into adult BM CD34^+^ cells, IGF2BP1 was re-expressed in adult BM CD34^+^ cells, suggesting that epigenetic modifications may be responsible for the silencing of IGF2BP1 in adult cells (Ioannidis et al. 2005).

Some evidence showed that RNA binding proteins have a nonnegligible role in β-hemoglobin disorders. LIN28B and IGF2BP1 are known posttranscriptional regulators that depress the translation of BCL11A, thereby influencing HbF production (Basak et al. 2020; de Vasconcellos et al. 2017). Recently, another RNA-binding protein, RBM12, has been identified as a novel suppressor of HbF (Wakabayashi et al. 2022). RBM12 contains five RRM domains, but only the first one is essential for HbF regulation (Wakabayashi et al. 2022). Mechanically, some RNA-binding proteins promote HbF production, while some repress its expression. Whether there are more RNA-binding proteins involved in HbF regulation in the future remains to be explored.

At last, IGF2BP1 holds promise as a broad-spectrum tumor marker for early diagnosis and prognostic evaluation. Given the significant enhancement of IGF2BP1 on γ-globin production in β-thalassemia and SCD patients, we hope that small molecule agonists targeting IGF2BP1 can be developed in the future. Such advancements could offer new hope to the many patients suffering from β-globin disorders.

## Data Availability

Not applicable.

## Abbreviations

IGF2BP1: Insulin-like growth factor 2 binding protein 1
PTEN: Phosphatase and tensin homolog
ACTB: Actin beta
MAPK4: Mitogen-activated protein kinase 4
c-MYC: MYC proto-oncogene
CD44: CD44 molecule
m6A: N6-methyladenosine
TME: Tumor immune microenvironment
AML: Acute myeloid leukemia
ALL: Acute lymphoblastic leukemia
CML: Chronic myeloid leukemia
CLL: Chronic lymphocytic leukemia
IGF2BP3: Insulin-like growth factor 2 mRNA binding protein 3
IGF2BP2: Insulin-like growth factor 2 mRNA binding protein 2
RRMs: RNA recognition motifs
KH: hnRNP-K homology
CRD: c-MYC-coding region stability determinant
CNC: Cranial neural ridges
AMO: Antisense morpholino oligonucleotides
ITGA6: Integrin subunit alpha 6
UTR: Untranslated regions
CDS: Coding sequence
HbF: fetal hemoglobin
LCR: Locus control regions
SCD: Sickle cell anemia
HBB: adult hemoglobin
BM: Bone marrow
